# Evidence-based surgery for laparoscopic appendectomy: A stepwise systematic review

**DOI:** 10.1016/j.sopen.2021.08.001

**Published:** 2021-08-26

**Authors:** Kovi E. Bessoff, Jeff Choi, Christopher J. Wolff, Aditi Kashikar, Garrison M. Carlos, Luke Caddell, Rida I. Khan, Christopher D. Stave, David A. Spain, Joseph D. Forrester

**Affiliations:** a Division of General Surgery, Department of Surgery, Stanford University, Stanford, CA, USA; b Students and Surgeons Writing About Trauma, Department of Surgery, Stanford University, Stanford, CA, USA; c Digestive Diseases and Surgery Institute, Cleveland Clinic Akron General, Akron, OH; d Department of Surgery, Emory University School of Medicine, Atlanta, GA; e University of California Berkeley, Berkeley, CA; f Lane Medical Library, Stanford School of Medicine, Stanford, CA

## Abstract

**Introduction:**

Appendectomy is a common emergency surgery performed globally. Despite the frequency of laparoscopic appendectomy, consensus does not exist on the best way to perform each procedural step. We identified literature on key intraoperative steps to inform best technical practice during laparoscopic appendectomy.

**Methods:**

Research questions were framed using the population, indication, comparison, outcome (PICO) format for 6 key operative steps of laparoscopic appendectomy: abdominal entry, placement of laparoscopic ports, division of mesoappendix, division of appendix, removal of appendix, and fascial closure. These questions were used to build literature queries in PubMed, EMBASE, and the Cochrane Library databases. Evidence quality and certainty was assessed using Grading of Recommendations, Assessment, Development, and Evaluation (GRADE) definitions.

**Results:**

Recommendations were rendered for 6 PICO questions based on 28 full length articles. Low quality evidence favors direct trocar insertion for abdominal entry and establishment of pneumoperitoneum. Single port appendectomy results in improved cosmesis with unclear clinical implications. There was insufficient data to determine the optimal method of appendiceal stump closure, but use of a specimen extraction bag reduces rates of superficial surgical site infection and intra-abdominal abscess. Port sites made with radially dilating trocars are less likely to necessitate closure and are less likely to result in port site hernia. When port sites are closed, a closure device should be used.

**Conclusion:**

Key operative steps of laparoscopic appendectomy have sufficient data to encourage standardized practice.

## INTRODUCTION

Appendicitis is a common surgical emergency. Lifetime disease risk in the United States is approximately 8% and appears to be increasing in many low and middle Human Development-Index Countries (LMHDICs) [1]. Appendectomy has become one of the most commonly performed emergency abdominal operations since its description by McBurney in the 1890s [2]. Laparoscopic appendectomy (LA), first introduced in the early 1980s, is associated with shorter hospitalization and lower complication rates compared to open appendectomy for uncomplicated appendicitis [3,4]. Benefits of LA persist in high-risk patient populations (eg, obese and elderly patients and those with medical comorbidities) as well as in LMHDIC cohorts [5]. Multiple surgical societies now recommend LA as first-line treatment for uncomplicated appendicitis [3,4,6].

However, there remains incomplete consensus on the sequence and execution of key technical steps to optimize clinical outcomes and minimize resource utilization during LA. Critical evaluation of published literature for each step of an operation has been useful to standardize other common surgical procedures like cesarean delivery [7,8]. Our review aims to critically evaluate published literature examining key intraoperative steps of LA to identify best practices for each intraoperative step.

## METHODS

**Fig 1:**
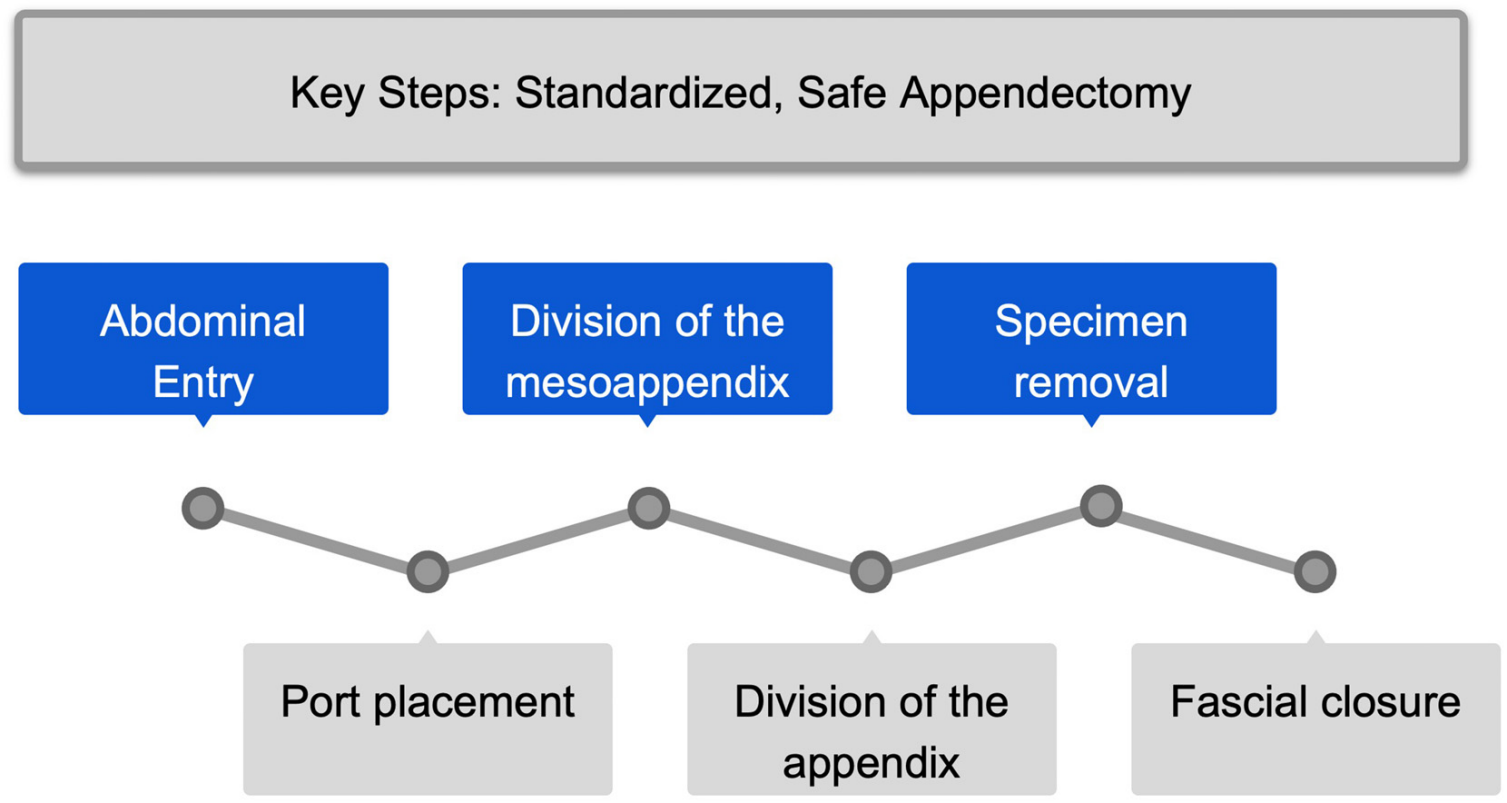
Key steps in laparoscopic appendectomy.

Research questions were framed using population, indication, comparison, outcome (PICO) format. We examined intraoperative steps of LA and did not examine other aspects of appendicitis management such as diagnosis, nonoperative management, or postoperative care. Six key operative steps were identified by consensus among authors (KB, JC, JF): (1) abdominal entry, (2) placement of laparoscopic ports, (3) division of mesoappendix, (4) division of appendix, (5) removal of appendix, and (6) fascial closure (Fig 1).

A research librarian (CS) designed and performed extensive searches of 3 bibliographic databases [PubMed (includes MEDLINE), EMBASE, and the Cochrane Library] for the 6 PICO questions corresponding to key intraoperative steps (Supplemental File 1). Eligible articles included experimental and observational studies in the English language published from database inception to April 10, 2020. We included studies evaluating adult (age ≥ 18 years) patients with a diagnosis of acute appendicitis. We excluded case reports, commentaries, reviews, and animal studies. Database results were uploaded to Covidence systematic review software (Veritas Health Innovation, Melbourne, Australia). Studies examining abdominal entry (PICO 1) and fascial closure (PICO 6) in the context of any laparoscopic procedure were included to augment the quality of the data available to answer these PICO questions.

Two authors (KB and JC) identified studies relevant to each PICO question. Only randomized controlled trials (RCTs) were included for a PICO question evaluated by ≥ 3 RCTs. Observational studies were included for PICO questions evaluated by < 3 RCTs. Disagreement was resolved by consensus. Additional articles discovered during further research were included in the analysis as appropriate. Evidence quality and certainty were assessed using the Grading of Recommendations, Assessment, Development, and Evaluation (GRADE) definition [9]. All studies were evaluated using a standardized data extraction template. This study did not meet criteria for IRB review because it was an analysis of previously published work.

## RESULTS

**Fig 2:**
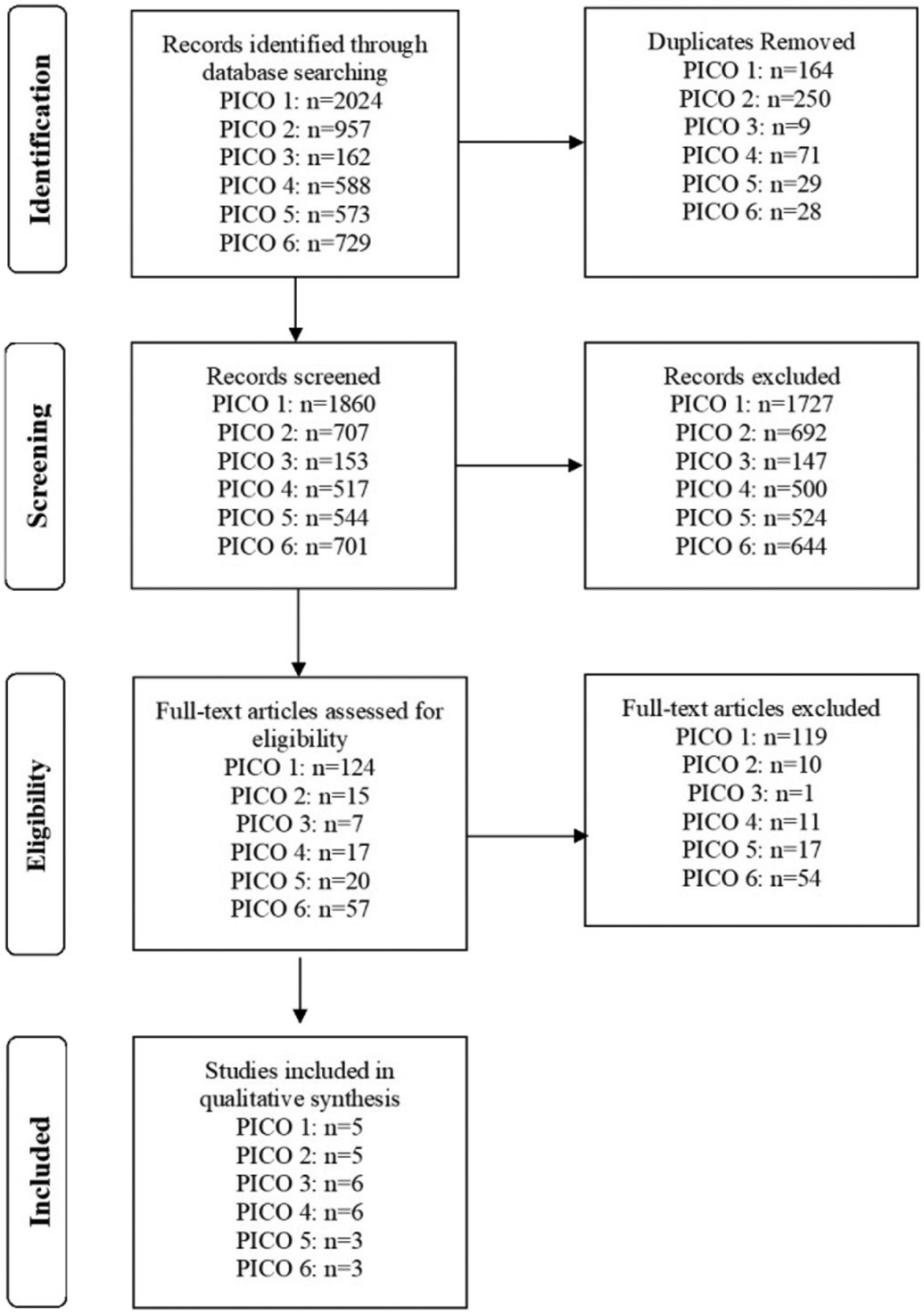
PRISMA flow diagram.

The PRISMA flow diagram is provided in Figure 2. We identified a total of 5,033 manuscripts, from which 28 full text articles underwent evidence synthesis (PICO 1: *n* = 5; PICO 2: *n* = 5; PICO 3: *n* = 6; PICO 4: *n* = 6; PICO 5: *n* = 3; and PICO 6: *n* = 3).

## PICO 1: In Adult Patients Undergoing LA for Uncomplicated Appendicitis, What Is the Best Method to Establish Pneumoperitoneum to Minimize Perioperative Morbidity?

### Background: Abdominal Entry

Approximately 50% of injuries sustained during laparoscopic abdominal surgery occur while establishing pneumoperitoneum [10]. Common approaches for abdominal access include use of direct trocar entry (DTE) with blunt or bladed trocars (trocar insertion prior to establishing pneumoperitoneum), Veress needle (VN) (blind insufflation), direct optical entry (DOE; visualizing initial trocar insertion through each layer of the abdominal wall), and Hasson technique (trocar insertion through larger incision and establishing pneumoperitoneum under direct visualization). Although this review primarily focuses on preventing perioperative morbidity, Supplemental Table 1 provides evidence-based recommendations related to efficiency and other outcomes. Of note, as techniques to establish pneumoperitoneum for lower abdominal and pelvic surgeries are similar, evaluated studies comprise laparoscopic operations beyond LA.

### Direct Trocar Entry Versus Veress Needle

One RCT evaluated complications after abdominal entry via DTE or VN among 1,000 gynecology patients undergoing diagnostic laparoscopy [11]. There were no differences in average body mass index, proportion of patients with prior abdominal surgeries, or indication for laparoscopy between groups. The study reported no major complications (vascular or visceral injury), but the incidence of minor complications (preperitoneal insufflation and omental emphysema) was higher in the VN group (10% and 4%, respectively) compared to the DTE group (0% and 0.4%, respectively; *P* < .001). The study reflected a single-surgeon experience and was not powered to capture minor complications.

*GRADE recommendation of study: Neither direct trocar entry nor Veress needle techniques resulted in any major complications within this study. Direct trocar entry did have a lower incidence of minor complications (GRADE: 2A).*

### Direct Trocar Entry Versus Hasson Technique

A single-center, noninferiority, RCT assessed complications following abdominal entry by DTE (*n* = 484) or Hasson technique (*n* = 478) in 955 patients with no abdominal surgical history undergoing laparoscopic general procedures [12]. There were no reported major vascular or solid organ injuries. Two major bowel injuries were reported in the Hasson group (unintentional enterotomy during cholecystectomy) compared to none in the DTE group (*P* = .1). There was an increase in umbilical port site infection (4.7% vs 1.0%; *P* < .01), umbilical port site pain (6.1% vs 1.2%; *P* < .01), and incidence of umbilical port site hernia (1.3% vs 0%; *P* < .01) in the Hasson group compared to the DTE group.

*GRADE recommendation of study: Direct trocar entry has a lower incidence of perioperative complications compared to the Hasson technique for abdominal entry in laparoscopic surgery in patients with no history of abdominal surgery (GRADE: 1B).*

### Direct Optical Entry Versus Veress Needle

DOE with a bladeless trocar was compared to VN entry in nonobese, reproductive-aged women (DOE, *n* = 93; VN, *n* = 101) [13] and postmenopausal women (DOE, *n* = 89; VN, *n* = 87) [14] with no history of abdominal surgery undergoing laparoscopic cystectomy for simple ovarian cysts. No major vascular injuries were noted in either group, and there was no difference in minor vascular injury or blood loss. No bowel injuries were noted in the postmenopausal study, while two bowel injuries were reported in reproductive-aged women who underwent abdominal access by VN [a nonsignificant difference compared to the DOE group in this study (*n* = 0; *P* = .5)]. These studies considered a number of patient variables including age, body habitus, and surgical history; however, the concentration on gynecology patients may limit the generalizability of findings to male patients. Additionally, studies were underpowered to detect rare events.

*GRADE recommendation of study: There is no difference in perioperative complications between direct optical entry and Veress needle entry for abdominal access in laparoscopic surgery (GRADE: 2A).*

### Direct Optical Entry Versus Hasson Technique

Direct optical entry and Hasson technique were compared in normal-weight (DOE, *n* = 100; Hasson, *n* = 102) [13] and obese women (DOE, *n* = 108; Hasson, *n* = 116) [15] with no history of abdominal surgery, as well as nonobese women with a history of previous abdominal surgery (DOE, *n* = 86; Hasson, *n* = 102) [16] undergoing diagnostic laparoscopy for benign utero-ovarian disease. There were no reports of major or minor vascular injury or bowel injury in any cohort. Again, the study benefited from a wide array of patient variables; however, generalizability to male patients and patients with other pelvic pathology may be limited.

*GRADE recommendation of study: There is no difference in perioperative complications between direct optical entry and Hasson technique for abdominal access in laparoscopic surgery (GRADE: 2A).*

### Placement of Incision

In addition to techniques used to establish pneumoperitoneum, initial incision sites may affect subsequent surgical steps, potential for healing, pain, and cosmesis. Initial incisions around (periumbilical) and directly overlying (transumbilical) the umbilicus have been described. A meta-analysis of 5 RCTs (published 2016–2019) comprising 783 patients who underwent laparoscopic cholecystectomy, appendectomy, or gynecologic surgery found no difference in postoperative pain, surgical site infection, wound numbness or hypersensitivity, or hospital length of stay between patients with periumbilical and transumbilical incisions [17]. Evaluated studies had considerable heterogeneity in patient population and follow-up intervals, which were likely insufficient to adequately characterize cosmetic satisfaction and hernia incidence.

*GRADE recommendation of study: There is no difference in safety outcomes between transumbilical and periumbilical skin incisions during laparoscopy (GRADE: 1A).*

## PICO #1 Overall Recommendation

There is no significant difference in perioperative morbidity among direct optical entry, Hasson technique, and Veress needle, although lower-quality evidence favors direct trocar entry. Despite weak evidence suggesting that direct trocar entry may be marginally safer, it should be noted that this technique is rarely used in practice. There are no studies comparing direct trocar entry and direct optical entry. The location of the umbilical incision does not affect safety outcomes.

## PICO 2: In Adults Patients Undergoing LA for Uncomplicated Appendicitis, What Is the Preferred Port Placement Strategy to Optimize Surgical Outcomes?

### Background: Port Placement

In the absence of umbilical hernia or urachal anomaly, there are no critical structures at the umbilicus, making it the ideal location for port placement [18]. Traditional LA port placement includes periumbilical, left lower quadrant, and suprapubic ports. Several techniques involving single periumbilical incisions emerged in recent years (Table 1).

**Table 1:** Nontraditional port configurations for laparoscopic appendectomy

*Technique*	*Reference*	*Special equipment*	*Uses traditional instruments?*	*Fascial defects*	*Port placement*
SPILS	SCARLESS Study Group (2015)	Multichannel port	Yes	1	Multichannel port placed through a transumbilical skin incision
SILS	Carter et al (2014)	SILS port	Yes	1	Skin incision through umbilical stalk. SILS device placed through a 30-mm fascial defect
SPLA	Lee et al (2013)	Octoport wound retractor	Yes	1	15-mm transumbilical skin incision. Octoport placed through 25–30-mm fascial defect.
TSILA	Pan et al (2013)	None	Yes	2	15-mm transumbilical skin incision. 5- and 10-mm ports placed in close proximity through separate fascial defects. Appendix suspended from abdominal wall via a silk suture.
LESS	Teoh et al (2012)	None	Yes	3	13-mm transumbilical skin incision. Separate fascial incisions for two 5-mm and one 10-mm laparoscopic ports

#### Conventional 3-Port Laparoscopic Appendectomy Versus Single-Port/Incision Laparoscopic Surgery

The Scarless Study group randomized adults without a history of laparotomy presenting with acute appendicitis to undergo single-port/incision laparoscopic surgery (SPILS) (*n* = 39) or conventional LA (*n* = 38) [19]. Among patients assigned to the SPILS group, 2 required an additional port, 3 underwent conventional appendectomy, and 1 underwent open appendectomy. Among those assigned to conventional LA, 2 required additional ports and 2 underwent open appendectomy. Both groups had similar hospital length of stay, time to return to normal activities, and complication rates. However, the SPILS group had higher cosmetic scores (as measured by the Body Image Questionnaire) [18.9 (SD = 4.1) vs 15.3 (SD = 5.8); difference: 3.6 (95% CI 0.7–6.5); *P* = .016], higher body image scale scores [5.6 (SD = 1.0) vs 7.0 (SD = 3.3); difference: 1.4 (95% CI − 2.8 to 1.5); *P* = .03], and lower readmission rates (5% vs 18%, no *P* value provided). The study was designed to detect a 0.65 difference in body image questionnaire with 80% power but was likely underpowered to detect less common complications.

*GRADE recommendation of study: SPILS is associated with improved cosmesis and satisfaction, less pain, and lower readmission compared to conventional LA in adults with no history of abdominal surgery undergoing LA for acute appendicitis (GRADE: 2B).*

#### Conventional 3-Port Laparoscopic Appendectomy Versus Single-Incision Laparoscopic Surgery

A single-institution, nonblinded RCT randomized patients with acute appendicitis to undergo conventional LA or single-incision laparoscopic surgery (SILS) [20]. The study planned to enroll 150 patients but was halted after interim analysis (*n* = 75 patients) showed significantly higher mean pain scores 12 hours after surgery (primary outcome) in the SILS group (4.4 out of 10; SD ± 1.6) compared to the conventional laparoscopy group (3.5 out of 10; SD ± 1.5) (*P* = .01). Additionally, SILS group required more inpatient opioids (mean hydromorphone use: 3.9; SD ± 1.9 mg vs 2.8; SD ± 1.7 mg; *P* = .01) compared to conventional appendectomy patients. Both groups had similar complication rates (including wound infection, deep space infection, seroma, postoperative bleeding, urinary retention, and postoperative ileus) and 30-day readmission rates (5%: SILS, 3%: conventional, *P* = .61). At 6-month telephone follow-up, no patients reported port site hernia; the conventional LA group reported higher body image perception scores (4.0; SD ± 0.4 vs 3.8; SD ± 0.4, *P* < .01) but worse overall cosmetic impression of scars (16.4; SD ± 3.0 vs 18.4; SD ± 2.7, *P* < .01).

*GRADE recommendation of study: SILS results in increased postoperative pain compared to conventional 3-port appendectomy in adult patients undergoing appendectomy for acute appendicitis (GRADE: 1B).*

#### Conventional 3-Port Laparoscopic Appendectomy Versus Single-Port Laparoscopic Appendectomy

A single-institution RCT of adults with acute appendicitis without intra-abdominal abscess randomized patients to undergo conventional LA (*n* = 124) or single-port laparoscopic appendectomy (SPLA) (*n* = 124; 10% required an extra port) [21]. SPLA and conventional LA groups had similar overall complication rates (14.6% vs 17.7%; *P* = .47), median hospital length of stay [SPLA: 3 days (IQR 2–4) vs conventional LA: 3 days (IQR 2–5), *P* = 1.0], number of doses of oral analgesia (1.7 vs 1.8 doses; *P* = .41), need for rescue analgesia (1.3 vs 1.2 doses; *P* = .78), need for surgical drains (4.3% vs 7.1%; *P* = .37), mean cosmetic satisfaction scores (4.0 vs 3.3, *P* = .128), postoperative pain scores (at 12 hours, 24 hours, 36 hours, and 14 days), and 36-item short-form health survey quality of life scores (at 2 or 14 days postoperatively).

*GRADE recommendation of study: SPLA and conventional appendectomy have similar outcomes in adult patients undergoing LA for acute appendicitis (GRADE: 2A).*

#### Conventional 3-Port Laparoscopic Appendectomy Versus Transumbilical Single-Incision Laparoscopic Appendectomy (TSILA)

A single-institution RCT randomized nonpregnant patients aged > 16 years with acute appendicitis to undergo transumbilical single-incision laparoscopic appendectomy (TSILA) (*n* = 42) or conventional LA (*n* = 42) [22]. Two TSILA cases were converted to conventional LA. No port site hernias were reported, and both groups had similar hospital length of stay. Patients undergoing TSILA reported higher mean cosmesis scores (appearance of incision, pain, itchiness, stiffness, thickness, and irregularity; 4.5 vs 3.9; *P* < .001).

*GRADE recommendation of study: TSILA produces superior cosmetic outcomes to conventional laparoscopic appendectomy (GRADE: 2A).*

#### Conventional 3-Port Laparoscopic Appendectomy Versus Laparoendoscopic Single-Site Appendectomy

A two-institution RCT randomized patients with appendicitis to undergo laparoendoscopic single-site (LESS) appendectomy (*n* = 100) or conventional LA (*n* = 100) [23]. LESS and conventional LA groups had similar complication rates (14.3% vs 9.3%, *P* = .39), rate of conversion to open appendectomy (8.2% vs 3.1%, *P* = .26), and number of total oral analgesic doses [3.2 doses (SD = 3.5) vs 3.0 doses (SD = 5.35); *P* = .18]. Both groups also had similar operative time, hospital length of stay, time to return to regular diet/normal activity, and quality of life scores 2 weeks postoperatively. Cosmesis scores [82.5 (SD = 20.2): LESS, 73.4 (SD = 24.1): conventional appendectomy; *P* = .002] and satisfaction scores [86.2 (SD = 17.6): LESS, 80.0 (SD = 24.42): conventional appendectomy *P* = .05] were higher in the LESS group.

*GRADE recommendation of study: LESS leads to better cosmetic outcomes (GRADE: 2A).*

## PICO #2 Overall Recommendation

There appears to be a trend toward improved cosmesis and self-image scores with single-incision laparoscopic appendectomy compared to conventional 3-incision laparoscopic appendectomy. However, the clinical impact of incremental changes in cosmesis scores is not clear. Postoperative pain varies significantly between studies and techniques.

## PICO 3: In Adult Patients Undergoing LA for Uncomplicated Appendicitis, What Is the Best Method to Divide the Mesoappendix to Minimize Perioperative Morbidity (Significant Bleeding Requiring Transfusion or Reoperation) and Mortality?

### Background: Division of the Mesoappendix

Safe division of the mesoappendix is a critical step in LA that may be hindered by tissue edema and inflammation [24]. Endoscopic gastrointestinal anastomosis (endo-GIA) staplers have been shown to safely decrease operative time compared to the ligation with an endoloop device and may be a safe and efficient means to simultaneously divide the mesoappendix and the appendix proper [25]. Additionally, electrocoagulation devices including the LigaSure vessel sealing device and HARMONIC scalpel have been shown to be efficient and safe methods to divide the mesoappendix [26].

### LigaSure Versus Sharp Division Between Endoclips

Between August 2007 and June 2008, 32 patients undergoing LA for acute, uncomplicated appendicitis were randomized (1:1) to mesoappendix division by LigaSure versus sharp division between endoclips [24]. Whereas mean operative time was lower in the LigaSure group (49 min; SD ± 15 min vs 60 min; SD ± 13 min; *P* = 0.036), the authors found no difference between groups with respect to need for analgesia or hospital length of stay. No complications occurred in either group. This was a well-controlled study but was limited by small cohort size from a single institution, making it underpowered to detect rare complications. Additionally, granular data regarding complications were limited.

*GRADE recommendation of study: Either LigaSure or sharp division between endoclips can be used to safely divide the mesoappendix (GRADE: 2B).*

### Sharp Division Between Polymeric Clips Versus Endoscopic Stapler

A prospective cohort study examined 92 patients 6 years of age or older with acute appendicitis (73% uncomplicated) who underwent LA by 20 different surgeons at a single community hospital between June and September 2016 [27]. Eight surgeons (*n* = 45 patients) sharply divided the mesoappendix between polymeric (Hem-O-Lok), clips while the other 12 surgeons (*n* = 47 patients) used an endoscopic stapler to divide the structure. Propensity score matching was used to account for differences in baseline demographics between the 2 groups. Rates of postoperative complication (abdominal abscess, wound infection, ileus, and UTI) did not differ between groups (Hem-O-Lok: 2.6% vs endoscopic stapler: 10.5%, *P* = .17). Mean operative time, length of hospital stay, and incidence of intraoperative events did not differ between the groups in the propensity score–matched cohort. There was no difference in mean blood loss between the groups, but 3 patients in each group experienced intraoperative bleeding requiring the placement of metal clips or endoscopic stapling to achieve hemostasis. Two patients in the Hem-O-Lok group had thick, inflamed mesenteries, and the operating surgeon elected to use an endoscopic stapler instead of polymeric clips due to safety concerns. Study weaknesses include lack of formal randomization scheme, as well as low study numbers, rendering it underpowered to detect rare complications.

*GRADE recommendation of study: There is no difference in perioperative morbidity when the mesoappendix is divided sharply between polymeric clips or divided with an endoscopic stapler (GRADE: 2B).*

### Retrospective Studies

Several retrospective studies assessed optimal techniques for dividing the mesoappendix. Wright et al [28] conducted a retrospective review of 565 patients who underwent LA by 30 different surgeons for uncomplicated appendicitis between 2006 and 2011 at 2 university-affiliated hospitals in the United States. The mesoappendix was isolated from the appendix and taken with a linear stapler in 259 patients. In 149 patients, the mesoappendix and appendix were taken in a single fire of a linear stapler without dissection of the structures. Ultrasonic (HARMONIC) shears were used to divide the mesoappendix in the remaining 157 patients. There was no difference in hematoma or abscess formation, or the need for transfusion or reoperation between the groups [35].

Lee and Hong [29] reviewed 1178 patients with acute appendicitis (83.6% uncomplicated) treated in a Korean military hospital from January 2003 to April 2013. Endoclips were used to ligate the mesoappendix in 460 patients followed by sharp division with endoshears or coagulation using electrocautery. A HARMONIC scalpel was used to divide the mesoappendix in 372 patients, and monopolar cautery alone was used to take the mesoappendix in the remaining 346 patients. There was no difference in the incidence of wound infection, abscess formation, ileus, or hemorrhage between groups. Additionally, rates of conversion to open appendectomy and length of hospital stay did not vary significantly between groups.

Finally, Aydogan et al [30] examined a cohort of 280 patients who presented with acute appendicitis (75.7% uncomplicated) to a university-affiliated hospital in Turkey from May 2003 to April 2007. The mesoappendix was divided with a LigaSure vessel sealer (*n* = 127) or divided between endoclips (*n* = 153). There was no difference in the frequency of wound infection or intra-abdominal abscess between the groups. Overall, the rate of conversion to open appendectomy was higher in the endoclip group compared to the LigaSure group (11.1% vs 9.4%; *P* < .05). However, bleeding-related conversion was not different between groups.

*GRADE recommendation of study: Retrospective studies have not identified a standout mesoappendiceal division technique for mitigating bleeding or infectious complications (GRADE: 2C).*

## PICO #3 Overall Recommendation

Available data are insufficient to render a recommendation regarding the optimal technique to divide the mesoappendix during LA based on perioperative morbidity and mortality alone. Given the lack of convincing data, other considerations including operative efficiency and cost may be useful in determining the ideal technique for division of the mesoappendix.

## PICO 4: In Adult Patients Undergoing LA for Uncomplicated Appendicitis, What Is the Best Method to Divide the Appendix to Minimize Perioperative Morbidity (Appendiceal Stump Blowout, Abscess Formation, Need for Antibiotic Therapy, Need for Reoperation) and Mortality?

### Background: Division of the Appendix

Failure to close the appendiceal stump can result in infectious complications. Laparoscopic closure of the appendiceal stump with staplers and endoloops has been described [31]. However, closure with these devices may be prone to failure when the appendiceal base is very inflamed and friable [32], and surgeons have reported cases of loose staples acting as a nidus for adhesive disease and bowel obstruction [33,34]. Suture ligation with Vicryl, polyglactin, or polydioxanone suture has also been shown to be an effective method to ligate the appendiceal stump. The endoloop, a modified Roeder loop, is a preformed ligature that can be readily passed over the appendix and tightened around the base of the appendix [35]. The use of titanium endoclips was first described by Cristalli et al [36] and has been shown to be a safe and efficient means to close the appendiceal stump. Endoclips have the theoretical advantage of being biologically inert, minimizing the degree of subsequent inflammation [37].

### Endoclips Versus Suture Ligation

Ates et al conducted a prospective RCT in a single academic medical center in Turkey between April 2010 and February 2011 [38]. Sixty-one patients with acute appendicitis (59% female, 62% uncomplicated) underwent appendectomy with division of the appendix between endoclips (*n* = 30) or following intracorporeal ligation with a polyglactin suture (*n* = 31). There were no differences between rates of intraoperative complications (subcutaneous/preperitoneal emphysema, dropped clip, inferior epigastric artery injury, and conversion to open) or postoperative compilations (intra-abdominal abscess, surgical site infection, abdominal pain, or need for reoperation). Length of hospital stay did not differ between groups. This study cohort was small, and although the authors did perform a priori power calculations, it is not clear for which complications the study was powered. Mean follow-up (180 ± 82 days for the endoclip group and 175 ± 82 days for the intracorporeal ligation group) fell short of the 2-year median documented endoclip migration noted for other intra-abdominal procedures [39].

*GRADE recommendation of study: There is no difference in perioperative morbidity and mortality when endoclips or polyglactin suture ligation is used for appendiceal stump closure in adult patients undergoing LA for acute appendicitis (GRADE: 2B).*

### Single Versus Double Endoloop

Beldi et al [40] sought to determine the optimal number of endoloops to close the appendiceal stump. The authors conducted a single-center, prospective RCT at a single institution in Switzerland between July 1999 and November 2000 in 271 patients with acute appendicitis (93.8% uncomplicated). Patients were randomized to ligation of the appendiceal base with one (*n* = 109) or two (*n* = 99) polydioxanone endoloops. There was no difference in complication rates between groups. Of note, an endolinear stapler was used for all operations where there was concern for a necrotic base (*n* = 24), and 39 cases were converted to open appendectomy. These 63 cases were excluded from the final analysis, creating bias. Additionally, the study was underpowered to detect complications.

*GRADE recommendation of study: In patients undergoing LA for acute appendicitis without a necrotic base, appendiceal stump closure with a single or double endoloop found similar outcomes (GRADE: 2B).*

### Polymeric Clips Versus Endoloop

A prospective, single-center, randomized controlled trial compared double nonabsorbable polymeric clips to double endoloop ligature for closure of the appendiceal stump in 60 nonpregnant patients over the age of 16 years with acute appendicitis (15% complicated appendicitis) at an academic medical center in Turkey between September 2010 and July 2011 [35]. There was no statistically significant difference between groups with respect to hospital length of stay, surgical site infection, intra-abdominal abscess, or nonsurgical complications. This was a small, single-site study that was underpowered to detect rare complications with a short follow-up interval (4 weeks). The authors failed to use an intent-to-treat methodology for analysis, excluding 7 patients who required conversion to open appendectomy and 4 patients who were lost to follow-up.

*GRADE recommendation of study: This study does not provide adequate data to support a recommendation for appendiceal stump closure.*

### Endostapler, Endoloop, Polymeric Clips, and Titanium Clips

A prospective, randomized study compared outcomes of stump closure in 120 patients with acute appendicitis (49% complicated appendicitis) using single endoloop ligature (*n* = 30), nonabsorbable polymeric clip (*n* = 30), titanium clip (*n* = 30), or a 45-mm load of an endostapler (*n* = 30) [41]. There were no recorded complications in the 30-day follow-up window, and overall length of stay did not vary between groups. The study suffered from single-center design, lack of a priori power calculations, and a low sample size per trial arm. This resulted in its being underpowered to detect perioperative complications, as suggested by the absence of any complications.

*GRADE recommendation of study: This study does not provide adequate data to support a recommendation for appendiceal stump closure.*

### Suture Ligation Versus Titanium Endoclips

Nadeem et al [42] undertook a prospective, single-blinded, multicenter randomized controlled trial from June 2013 to June 2014 at 3 tertiary care hospitals in Pakistan. Sixty-eight patients with clinically diagnosed uncomplicated appendicitis (Alvarado score ≥ 8) were randomized to LA with closure of the appendiceal stump by titanium endoclips (*n* = 32) or suture ligation with a 0-Vicryl suture fashioned into a Roeder knot (*n* = 36). There was no difference in frequency of intraoperative compilations (bleeding and visceral injury) or postoperative complications (ileus, intra-abdominal infection, surgical site infection), reoperation rate, or readmission rate. This study examined a relatively small cohort of patients limited to uncomplicated appendicitis, making it underpowered to detect rare complications and limiting generalizability of findings. Additionally, interventions were assigned at the discretion of the operating surgeon, and data collection was not performed in a blinded manner, increasing the risk of bias.

*GRADE recommendation of study: This study does not provide adequate data to support a recommendation for appendiceal stump closure.*

#### Closure With Endoloop Versus Endoclips Following Division of Appendix With Vessel Sealer

Sadat-Safavi et al [43] conducted a prospective RCT of 76 patients with acute appendicitis who underwent LA by a single surgeon in Iran from March 2013 to May 2015. Following division of the mesoappendix with a LigaSure device, patients were randomized to closure of the appendiceal stump with endoclips (*n* = 38, 18 male, 22 ± 3.69 years old) or endoloop (*n* = 38, 16 male, 24.3 ± 6.0 years old). There were no statistically significant differences with respect to the frequency of wound infection, surgical site pain, technical compilations, stump leak, the need for reoperation, or length of hospital stay. Again, this study was underpowered to detect rare complications associated with appendectomy, and there was little discussion of the study design including blinding and the randomization process.

*GRADE recommendation of study: This study does not provide adequate data to support a recommendation for appendiceal stump closure.*

## PICO #4 Overall Recommendation

Among 6 RCTs examining different techniques for appendiceal stump closure, no study found a statistically significant difference in perioperative complication rates. There is insufficient evidence supporting a standout technique for appendiceal stump closure. Supply availability, familiarity, and costs may help guide surgeon choice.

## PICO 5: In Adult Patients Undergoing LA for Uncomplicated Appendicitis, What Is the Best Method to Extract the Divided Appendix From the Abdomen to Minimize Perioperative Morbidity (Intra-Abdominal Abscess, Surgical Site Infection)?

### Background: Specimen Removal

Optimal techniques to reduce superficial (surgical site infection; SSI) and deep space (intra-abdominal abscess; IAA) perioperative infection associated with appendectomy are an ongoing topic of debate. Meta-analyses suggest that although LA decreases the risk of SSI, the approach may lead to increased incidence of IAA (compared to open appendectomy) [44,45], whereas an international, multicenter retrospective review found that IAA rate was lower in patients with complicated appendicitis who were treated with LA [46]. Laparoscopic specimen retrieval bags have been explored as a means to decrease the rate of perioperative infection during LA.

### Specimen Retrieval Bag Versus Direct Extraction

Using the 2016 American College of Surgeons National Surgical Quality Improvement Program (ACS NSQIP) Procedure Targeted Appendectomy database, Fields et al [47] retrospectively analyzed the postoperative course of 11,475 patients who underwent LA. A specimen retrieval bag was used to remove the appendix in 92% (*n* = 10578) of cases. The overall incidence of SSI and IAA was 0.9% and 2.8%, respectively. There was no difference in SSI between groups (0.6% vs 0.9%, *P* = .28). Multivariate analysis found the use of specimen retrieval bags to be associated with lower odds of IAA formation (OR 0.6, 95% CI 0.42–0.95, *P* = .03). There was no difference between groups with respect to the incidence of sepsis/septic shock (3.9% vs 3.3%, *P* = .26), need for reoperation (1.3% vs 1.0%, *P* = .32), or readmission (3.6% vs 3.8%, *P* = .77). This study benefited from a large patient cohort but was retrospective in nature and based on a single year of retrospective data which could not be adjusted for antibiotic use.

*GRADE recommendation of study: The use of a specimen retrieval bag is associated with decreased risk of IAA formation in adult patients undergoing LA (GRADE: 1C).*

Turner et al [48] also queried the 2016 Appendectomy-Targeted ACS NSQIP database for patients who had undergone LA. The authors limited their inclusion criteria to pathologically confirmed appendicitis and analyzed 10,357 patient records. Specimen retrieval bags were used in 93% (9,585) of cases. Overall infection rate (combined SSI and IAA) was 3.6% (*n* = 374; 89 with superficial SSI, 276 with IAA, and 9 with both SSI and IAA). Multivariable analysis did not identify significant difference in odds of perioperative complication 1.15 (CI 0.78–1.69; *P* = .49). Patients were further stratified based on whether they had complicated or uncomplicated appendicitis. This subgroup analysis did not reveal any difference in the incidence SSI or IAA. This study benefited from a large patient cohort. However, it was a temporally limited (data from a single year), retrospective study. Additionally, analysis was limited in that the authors chose to combine superficial (SSI) and deep space infections (IAA).

*GRADE recommendation of study: The use of a specimen retrieval bag does not influence the incidence of perioperative infection in adult patients undergoing LA (GRADE: 2C).*

Agalar et al [49] conducted a retrospective analysis of 213 patients with acute appendicitis treated with LA (54% female, median age 33.5 ± 13.8 years old, 23% complicated appendicitis) at a Turkish academic medical center from January 1, 2015, to January 1, 2017. In 112 patients, the specimen was removed using a retrieval bag, whereas the appendix was directly removed through a reusable trocar in the remainder (*n* = 101). Five patients in the direct removal group developed SSI compared to 0 in the retrieval bag group (*P* = .02). Three patients developed IAA, but there was no differentiation regarding mode of specimen extraction among these patients. Mean hospital length of stay was significantly longer in patients who developed surgical site infections (7.8; SD ± 3.96 days vs 2.8; SD ± 1.69 days, *P* < .01). This study was limited by its retrospective design and small incidence of complications (SSI *n* = 5; IAA *n* = 3).

*GRADE recommendation of study: In adult patients undergoing LA, using a specimen retrieval bag may be associated with lower risk of SSI (GRADE: 2C).*

## PICO #5 Overall Recommendations

Retrospective observational evidence suggests that use of a specimen extraction bag may be associated with lower risk of IAA and SSI. We recommend additional meta-analyses and/or a multicenter study to further strengthen these recommendations with particular attention to the cost–benefit of the use of additional equipment to prevent perioperative infection.

## PICO Question 6: In Adult Patients Undergoing LA for Uncomplicated Appendicitis, What Is the Best Fascial Closure Method to Minimize the Risk of Port Site Hernia/Other Complications?

### Background: Fascial Closure

Trocar site hernia is a relatively rare complication; reported incidence ranges from 0.02% to 3.6% [50]. These defects are often small, are difficult to detect, and produce few symptoms, leading to underreporting [50,51]. Dilating trocars are believed to result in a smaller fascial defect compared to cutting trocars [52]. Many suggest that fascial defects ≥ 10 mm require primary closure with [50] primary closure methods including use of a swedged needle, Carter–Thomason suture passer, or the neoClose device [53].

### Dilating Versus Cutting Trocars

Bhoyrul et al [54] conducted a multi-institutional RCT and randomized patients undergoing common laparoscopic general surgery procedures to blind entry using cutting (*n* = 125) or radially dilating (*Step*) trocar systems (*n* = 119) following establishment of pneumoperitoneum with a Veress needle. Surgeons were instructed to close defects > 10 mm (defined as accommodating the surgeon's pinky finger). No port site hernias were reported at 18-month follow-up. Only 3% in the dilating trocar group underwent trocar site primary closure, whereas 93% in the cutting trocar group underwent primary closure. The cutting trocar group had a higher incidence of minor intraoperative abdominal wall bleeding (10.6% vs 0%, *P* = .001), but there was no difference in the rate of major visceral or vascular injuries between the 2 groups.

*GRADE recommendation of study: Radially dilating trocars leads to decreased minor intraoperative abdominal wall bleeding and fewer fascial defects requiring primary closure compared to cutting trocars in adult patients undergoing laparoscopic surgery (GRADE: 2B).*

A single-center RCT randomized 56 patients undergoing laparoscopic surgery to placement of a 12-mm pyramidal blade cutting trocar (43 sites), single-blade cutting trocar (41 sites), axially dilating trocar (38 sites), or radially dilating *Step*-system trocar (43 sites) [55]. All trocars were inserted after establishing pneumoperitoneum with a Veress needle, and a lateral 5-mm, noncutting, metal trocar was placed in each case as a reference for pain scores. There was no statistically significant difference in mean pain scores at 3 hours, 24 hours, and 1 week among any group. The authors did not observe hernias at trocar sites despite not closing fascia in 82% of patients who had *Step* trocars placed. This was a well-designed study that sought to normalize pain scores and included a reference 5-mm lateral port site. Limitations included a relatively small patient cohort which was underpowered to detect hernias. Additionally, the follow-up was limited (3 months by telephone), which may limit diagnosis of small trocar site hernias.

*GRADE recommendation of study: Twelve-milliliter axially and radially dilating trocars decrease need for fascial closure in adult patients undergoing laparoscopic surgery without increasing postoperative pain (GRADE: 2B).*

### Suture Closure of Port Site Fascial Defects

Lago et al [50] set out to challenge the conventional wisdom that all trocar sites 10 mm and larger require fascial closure. The authors randomized 162 patients undergoing laparoscopic general surgery procedures (the majority, 79.6%, of which were appendectomy and cholecystectomy) by 1 of 5 surgeons at a Spanish academic medical center to closure of the external fascia with a #1 PDS suture or no closure. Following surgery, patients were screened by telephone at 1 and 2 years and asked about the development of lumps or pain at the incision site. Patients who screened positive were evaluated in person and examined by a surgeon for evidence of port site hernia. The authors only studied port sites that had not been dilated for specimen extraction or otherwise heavily manipulated. There was no statistically significant difference in the rate of hernia formation between the suture closure and no closure groups (5.0% vs 1.2%; *P* = .18), nor was there a significant difference in the rate of wound infection between these groups (8.8% vs 3.7%; *P* = .15). There was no difference in the rate of complications between the different attending surgeons. This was a well-designed, single-blinded, randomized prospective study with a good follow-up interval (average of 2 years and 3 months). Weaknesses of the study included strict exclusion criteria, particularly the decision to exclude port sites that had been manipulated, which may limit generalizability of the study findings. Additionally, the study was underpowered to detect hernias.

*GRADE recommendation of study: Suture closure of fascial defects may not prevent port site hernia in adult patients undergoing laparoscopic general surgery procedures (GRADE: 2B).*

### Hand-Sewn Versus Carter–Thomason Suture Closure

Primary port site fascial closure can be achieved by hand sewing or using specialized devices. Shetty et al [56] conducted a prospective controlled trial of 200 patients undergoing left laparoscopic donor nephrectomy at a single academic medical center in India. All patients had two 10-mm and two 5-mm trocars placed following establishment of pneumoperitoneum. One hundred consecutive patients (42 female; mean BMI 24.3 kg/m^2^) underwent hand-sewn fascial closure with a 2-0 Vicryl, and the second hundred consecutive patients (53 female; BMI 26.0 kg/m^2^) underwent fascial closure with a Carter–Thomason device. The authors observed more seromas (10% vs 1%; *P* = .005) and ascitic leaks (6% vs 0%; *P* = .012) in the hand-sewn closure group compared to the Carter–Thomason group. There were 2 wound infections noted in the hand-sewn group compared to 1 in the Carter–Thomason group, as well as a single incidence of incarcerated hernia in the hand-sewn group.

*GRADE recommendation of study: Use of a Carter–Thomason suture passer decreases the incidence of perioperative complication in patients undergoing abdominal laparoscopy (GRADE: 2B).*

### neoClose Device Versus Carter–Thomason for Fascial Closure

Iranmanesh et al [57] studied the efficacy of the neoClose device compared to traditional (Carter–Thomason) suture passer for closing fascial defects in obese patients undergoing robot-assisted laparoscopic weight loss surgery performed by 7 minimally invasive fellowship-trained surgeons at a single academic medical center in the United States from February 2016 to April 2018. Seventy patients undergoing bariatric surgery (robot-assisted sleeve gastrectomy or Roux-en-Y gastric bypass surgery) were randomized to fascial closure by neoClose device (*n* = 35) or traditional suture passer (*n* = 35). Patients were excluded if they had undergone previous laparotomy, weight loss surgery, or an abdominal hernia or if they required dilation of the trocar site to facilitate specimen extraction. Although no port site hernias were observed in either group at 12 months, this single-blinded, randomized prospective study had some significant limitations. The primary end point was difference in operative time and was therefore underpowered to detect port site hernia formation, and there was a high dropout rate. Twenty-three patients in the neoClose group and 24 patients in the traditional group were lost to follow-up by 12 months following surgery. Exclusion of patients requiring dilation of the port site for specimen extraction may limit applicability.

*GRADE recommendation of study: There is no difference in port site hernia formation following closure with the neoClose device or traditional Carter–Thomason suture passer in obese patients undergoing laparoscopic surgery (GRADE: 2C).*

## PICO #6 Overall Recommendations

The use of dilating trocars minimizes perioperative complications involving the abdominal wall as well as the frequency with which fascial defects need to be closed. When fascial closure is necessary, a suture passer or other closure device provides superior outcomes to hand-sewn closure.

In conclusion, laparoscopic appendectomy constitutes one of the most common procedures performed globally. Evidence-guided standardization and optimization of operative steps provide a unique opportunity to improve surgical outcomes for a large number of patients. Our systematic evaluation of the literature sought to review the data informing best practices in LA and make recommendations for additional research where necessary. Table 2 contains a summary of our findings, recommendations, and areas for additional research.

**Table 2:** PICO questions and conclusions

*PICO question*	*Recommendation*
1. In adults undergoing LA for uncomplicated appendicitis, what is the best method to establish pneumoperitoneum to minimize perioperative morbidity?	There is no significant difference in perioperative morbidity among direct optical entry, Hasson technique, and Veress needle, although lower-quality evidence favors direct trocar entry.
2. In adults undergoing LA for uncomplicated appendicitis, what is the preferred port placement strategy to optimize surgical outcomes?	Single port provides improved cosmesis, although the clinical implications of these changes are unclear.
3. In adults undergoing LA for uncomplicated appendicitis, what is the best method to divide the mesoappendix to minimize perioperative morbidity (significant bleeding requiring transfusion or reoperation) and mortality?	Insufficient data to make a recommendation
4. In adults undergoing LA for uncomplicated appendicitis, what is the best method to divide the appendix to minimize perioperative morbidity (appendiceal stump blowout, abscess formation, need for antibiotic therapy, need for IR, or reoperation) and mortality?	Insufficient data to make a recommendation
5. In adults undergoing LA for uncomplicated appendicitis, what is the best method to extract the divided appendix from the abdomen to minimize perioperative morbidity (intra-abdominal abscess, surgical site infection)?	The use of a specimen extraction bag decreases the risk of IAA and SSI
6. In adults undergoing LA for uncomplicated appendicitis, what is the best fascial closure method to minimize the risk of port site hernia/other complications?	The use of dilating trocars leads to fewer perioperative complications involving the abdominal wall and reduces the need for port site closure. Port site closure with a suture passer or other device results in superior outcomes.

The following are the supplementary data related to this article.

Supplemental File 1 Search strategy used for each PICO question Supplemental File 1

Supplemental Table 1 Evidence-based recommendations for efficiency of abdominal entry Supplemental Table 1

## Author Contributions

Drs Bessoff and Choi conceived the study, critically reviewed the literature, and participated in writing and editing the manuscript.

Drs Wolff, Kashikar, Carlos, and Caddell and Ms Khan critically reviewed the literature and participated in writing the manuscript.

Dr Choi and Mr Stave developed and executed the search strategy.

Drs Spain and Forrester assisted with the conceptual design of the study and provided critical analysis and editing.

## Conflict of Interest

The authors have no conflicts to declare.

## Funding Sources

No funding was received for the preparation of this manuscript.

## Funding/Financial Support

Authors received no funding/financial support associated with this work.
