# Supplementary material for: Evidence-based surgery for laparoscopic appendectomy: A stepwise systematic review
Source: Surg Open Sci. 2021 Aug 26;6:29–39. doi: 10.1016/j.sopen.2021.08.001 (PMC8473533; doi:10.1016/j.sopen.2021.08.001)
Supplement: Supplemental File 1 — Search strategy used for each PICO question [file mmc1.docx]

**Supplemental File 1:** Search strategy used for each PICO question

**PICO Question 1**

Pubmed (verres [tw] OR hasson [tw] OR "Pneumoperitoneum, Artificial/instrumentation"[Mesh] OR "Pneumoperitoneum, Artificial/methods"[Mesh] OR (Pneumoperitoneum, Artificial [mesh] AND "laparoscopy" [mesh])) AND english [lang] NOT ("animals" [mesh] NOT humans [mesh]) NOT Robotic Surgical Procedures [mesh] NOT robot* [ti] NOT letter [pt] NOT case reports [pt]

EMBASE (verres:ti,kw,ab OR hasson:ti,kw,ab OR 'artificial pneumoperitoneum'/exp) AND 'laparoscopy'/exp NOT 'robotic surgical procedure'/exp AND [humans]/lim AND [english]/lim AND ([embase]/lim NOT ([embase]/lim AND [medline]/lim) OR ([embase classic]/lim NOT ([embase classic]/lim AND [medline]/lim)))

Cochrane (verres OR hasson OR Pneumoperitoneum) AND laparoscopy AND (method* OR instrumentation OR technique* OR approach*)

**PICO Question 2**

Pubmed (("Appendectomy"[Mesh] AND "Laparoscopy"[Mesh]) OR “laparoscopic appendectom*” [tw] OR (laparoscop* [tw] AND appendectom* [tw])) AND (port [tw] OR ports [tw]) AND english [lang] NOT ("animals" [mesh] NOT humans [mesh]) NOT Robotic Surgical Procedures [mesh] NOT robot* [ti] NOT letter [pt] NOT case reports [pt]

EMBASE (("Appendectomy"/exp AND "Laparoscopy"/exp) OR “laparoscopic appendectomy*”:ti,ab,kw OR (laparoscop*:ti,ab,kw AND appendectom*:ti,ab,kw)) AND (port:ti,ab,kw OR ports:ti,ab,kw) NOT 'robotic surgical procedure'/exp AND [humans]/lim AND [english]/lim AND ([embase]/lim NOT ([embase]/lim AND [medline]/lim) OR ([embase classic]/lim NOT ([embase classic]/lim AND [medline]/lim)))

Cochrane laparoscop* AND appendectom* AND (port OR ports)

**PICO Question 3**

Pubmed (("Appendectomy"[Mesh] AND "Laparoscopy"[Mesh]) OR “laparoscopic appendectom*” [tw] OR (laparoscop* [ti] AND appendectom* [ti])) AND English [lang] AND (mesoappendix [tw] OR mesentery [tw] OR mesentery [mesh]) AND english [lang] NOT ("animals" [mesh] NOT humans [mesh]) NOT Robotic Surgical Procedures [mesh] NOT robot* [ti] NOT letter [pt] NOT case reports [pt]

EMBASE (("Appendectomy"/exp AND "Laparoscopy"/exp) OR “laparoscopic appendectomy*”:ti,ab,kw OR (laparoscop*:ti,ab,kw AND appendectom*:ti,ab,kw)) AND (mesoappendix:ti,ab,kw OR mesentery:ti,ab,kw OR “mesentery”/exp) NOT 'robotic surgical procedure'/exp AND [humans]/lim AND [english]/lim AND ([embase]/lim NOT ([embase]/lim AND [medline]/lim) OR ([embase classic]/lim NOT ([embase classic]/lim AND [medline]/lim)))

Cochrane laparoscop* AND appendectom* AND (mesentery OR mesoappendix)

**PICO Question 4**

Pubmed (("Appendectomy"[Mesh] AND "Laparoscopy"[Mesh]) OR “laparoscopic appendectom*” [tw] OR (laparoscop* [ti] AND appendectomy* [ti])) AND (divid* [tw] OR divisi* [tw] OR ligasure [tw] OR endoloop [tw] OR bipolar [tw] OR staple* [tw] OR electrocauter* [tw] OR cauter* [tw]) AND english [lang] NOT ("animals" [mesh] NOT humans [mesh]) NOT Robotic Surgical Procedures [mesh] NOT robot* [ti] NOT letter [pt] NOT case reports [pt]

EMBASE (("Appendectomy"/exp AND "Laparoscopy"/exp) OR “laparoscopic appendectomy*”:ti,ab,kw OR (laparoscop*:ti,ab,kw AND appendectomy*:ti,ab,kw)) AND (divid*:ti,ab,kw OR divis*:ti,ab,kw OR ligasure*:ti,ab,kw OR endoloop*:ti,ab,kw OR bipolar:ti,ab,kw OR staple*:ti,ab,kw OR electrocauter*:ti,ab,kw OR cauter*:ti,ab,kw) NOT 'robotic surgical procedure'/exp AND [humans]/lim AND [english]/lim AND ([embase]/lim NOT ([embase]/lim AND [medline]/lim) OR ([embase classic]/lim NOT ([embase classic]/lim AND [medline]/lim)))

Cochrane laparoscop* AND appendectom* AND (divid* OR divis* OR ligasure* OR endoloop* OR bipolar OR staple* OR electrocauter* OR cauter*)

**PICO Question 5**

Pubmed (("Appendectomy"[Mesh] AND "Laparoscopy"[Mesh]) OR “laparoscopic appendectom*” [tw] OR (laparoscop* [ti] AND appendectom* [ti])) AND (remov* [tw] OR retriev* [tw] OR endocatch* [tw] OR endobag* [tw]) AND english [lang] NOT ("animals" [mesh] NOT humans [mesh]) NOT Robotic Surgical Procedures [mesh] NOT robot* [ti] NOT letter [pt] NOT case reports [pt]

EMBASE (("Appendectomy"/exp AND "Laparoscopy"/exp) OR “laparoscopic appendectomy*”:ti,ab,kw OR (laparoscop*:ti,ab,kw AND appendectom*:ti,ab,kw))

AND (endobag*:ti,ab,kw OR remov*:ti,ab,kw OR retriev*:ti,ab,kw OR endocatch:ti,ab,kw) NOT 'robotic surgical procedure'/exp AND [humans]/lim AND [english]/lim AND ([embase]/lim NOT ([embase]/lim AND [medline]/lim) OR ([embase classic]/lim NOT ([embase classic]/lim AND [medline]/lim)))

Cochrane laparoscop* AND appendectom* AND (endobag* OR remov* OR retriev* OR endocatch)

**PICO Question 6**

Pubmed ("Laparoscopy"[Mesh] OR laparoscop* [tw]) AND (fasciotomy [mesh] OR fascia [tw] OR fascial [tw] OR “fascia” [mesh]) AND (closure [tw] OR "suture techniques" [mesh] OR port [ti] OR "incision site" [tw] OR “skin closure” [tw] OR “Wound Closure Techniques” [mesh]) AND english [lang] NOT ("animals" [mesh] NOT humans [mesh]) NOT Robotic Surgical Procedures [mesh] NOT robot* [ti] NOT letter [pt] NOT case reports [pt]

EMBASE ("Laparoscopy"/exp OR laparoscop*:ti,ab,kw) AND (“fascia”/exp OR fascia*:ti,ab,kw OR 'fasciotomy'/exp) AND (closure:ti,ab,kw OR "suture technique"/exp OR port:ti OR "incision site":ti,ab,kw OR “skin closure”:ti,ab,kw OR “Wound Closure”/exp) NOT 'robotic surgical procedure'/exp AND [humans]/lim AND [english]/lim AND ([embase]/lim NOT ([embase]/lim AND [medline]/lim) OR ([embase classic]/lim NOT ([embase classic]/lim AND [medline]/lim)))

Cochrane laparoscop* AND (facia* OR fascio*) AND (closure OR "suture techniques" OR port OR "incision site" OR “skin closure”)
