# Supplementary material for: Evidence-based surgery for laparoscopic appendectomy: A stepwise systematic review
Source: Surg Open Sci. 2021 Aug 26;6:29–39. doi: 10.1016/j.sopen.2021.08.001 (PMC8473533; doi:10.1016/j.sopen.2021.08.001)
Supplement: Supplemental Table 1 — Evidence-based recommendations for efficiency of abdominal entry [file mmc2.docx]

| **Reference** | **Treatment Groups** | **Findings** |
| --- | --- | --- |
| Time to peritoneal entry |  |  |
| Bemelman et al. (2000) | TrocDoc, Veress needle, Hasson technique | TrocDoc faster time to pneumoperitoneum (138 +/- 58 sec) compared to Veress (237 +/- 56 sec) and Hasson (350 +/- 103 sec) |
| Zakharea et al. (2010) | Direct trocar, Veress needle | Direct trocar faster time to pneumoperitoneum (2.2 +/- 0.7 min) compared to Veress needle (8.2 +/- 1.4 min) |
| Kaistha et al. (2019) | Direct trocar, Veress needle | Direct trocar faster mean access time to peritoneum (80 +/- 20 sec) compared to Hasson technique (181 +/- 27 sec) |
| Shih et al. (2020) | Transumbilical vs. periumbilical skin incision | Transumbilical incision lead to faster operative times compared to periumbilical incision (mean difference 7.7 minutes) |
| Efficiency of movement |  |  |
| Bemelman et al. (2000) | TrocDoc, Veress needle, Hasson technique | Veress needle required fewer movements (21.9 +/- 7) to achieve pneumoperitoneum compared to TrocDoc (31.9 +/-12) and Hasson technique (53 +/- 17) |
| Zakharea et al. (2010) | Direct trocar, Veress needle | Direct trocar group had fewer entries requiring multiple attempts (2%; 95%CI 0.8-3.2%) compared to Veress needle (14%; 95%CI 11.0-17.0%) |
| Use of gas |  |  |
| Zakharea et al. (2010) | Direct trocar, Veress needle | Direct trocar used less gas (2.6 +/- 0.9L) compared to Veress needle (8.4 +/- 2.6 L) |
| Kaistha et al. (2019) | Direct trocar, Veress needle | Direct trocar minimized gas leakage (0.8%) compared to Hasson technique (8.3%) |
